# In Vitro Profile of Hydrocortisone Release from Three-Dimensionally Printed Paediatric Mini-Tablets

**DOI:** 10.3390/pharmaceutics16030385

**Published:** 2024-03-11

**Authors:** Chrystalla Protopapa, Angeliki Siamidi, Siva Satyanarayana Kolipaka, Laura Andrade Junqueira, Dennis Douroumis, Marilena Vlachou

**Affiliations:** 1 Department of Pharmaceutical Technology, School of Pharmacy, National and Kapodistrian University of Athens, 157 84 Athens, Greece; cprotopapa@pharm.uoa.gr (C.P.); asiamidi@pharm.uoa.gr (A.S.); 2 Centre for Research Innovation, University of Greenwich, Medway Campus, Chatham Maritime, Chatham ME4 4TB, UK; s.kolipaka@gre.ac.uk (S.S.K.); ld3353f@gre.ac.uk (L.A.J.)

**Keywords:** hydrocortisone, 3D printing, micro-extrusion, direct powder extrusion, paediatrics, drug formulation development, pharmaceutical technology, personalized medicine, Gelucire^®^ 44/14, Precirol^®^ ATO 5

## Abstract

Three-dimensional (3D) printing is quickly being adopted in pharmaceutics due to the many advantages it offers, including treatment, adaptability, the reduction in waste and the accelerated development of new formulations. In this study, micro-extrusion printing was implemented for the production of modified-release hydrocortisone (HCT) mini-tablets for paediatric patients. For the developed formulations, Gelucire^®^ 44/14 and Precirol^®^ ATO 5 were used as the main inks at three different ratios: 70%/30%, 60%/40% and 50%/50%, respectively. The printing parameters (temperature and pressure) were altered accordingly for each ratio to achieve printability. The printed mini-tablets exhibited excellent printing quality, featuring consistent layer thicknesses and smooth surfaces. Dissolution tests were performed, and the results indicated a successful modified release of HCT from the mini-tablets. In summary, micro-extrusion exhibited favourable processing abilities for powder blends, facilitating quick printing and the fabrication of potential personalized dosages.

## 1. Introduction

In today’s society, social and technological developments are advancing rapidly, always with a focus on serving the needs of the people [1]. At the same time, current mass-manufacturing approaches to producing medicine are time-consuming, laborious and inflexible, resulting in medications being ineffective for approximately 70% of patients [2]. Amidst this substantial social and technological growth, it would be inconceivable not to harness new technologies for the achievement of a more personalized and effective treatment for patients. This can be accomplished through the utilization of 3D printers in formulation development [3]. With the increased availability of such tools, patient treatment is transitioning from traditional one-size-fits-all approaches to a wide range of dosage forms, designs and geometries [4].

The technology of 3D printing is quickly being adopted in pharmaceutics due to the many advantages it offers. The concept of ‘3D printing’ was first introduced in the early 1980s with the invention of stereolithography by Hideo Kodama [5]. The pursuit of prescribing more tailored medications according to individual patient conditions, genetic characteristics and physical states has become a shared goal among both patients and medical practitioners [6,7]. Yet, traditional pharmaceutical manufacturing processes often lack adaptability [8]. As an emerging technology in the realm of personalized medicine, 3D printing presents a high degree of adaptability, such as being able to adjust dosages or incorporate multiple drugs into a single dosage form, making it easier to administer combination therapies [9]. It offers precise control over the amount of each active pharmaceutical ingredient (API) in each dose, allowing for the creation of dosage forms with variable dosages in a single print run. Another advantage of 3D-printed medication is that it can increase patients’ compliance, especially in paediatric and geriatric populations, as dosage forms that are more patient-friendly in terms of shape, size and taste can be designed [10]. Moreover, pharmaceutical companies can use 3D printing to rapidly prototype new drug formulations, which can significantly speed up the development process and reduce time-to-market [11]. Additionally, 3D printing streamlines the production process, allowing for on-demand manufacturing and bolstering pharmaceutical efficiency, while simultaneously reducing waste, as 3D printing operates with only the necessary materials for each dosage form, compared to traditional pharmaceutical manufacturing, which often results in a substantial amount of waste due to the need for large batches and the use of excess materials [12]. In view of these benefits of 3D printing, substantial research effort has been invested in the realm of 3D printing over the past decade, leading to significant applications and advancements in the pharmaceutical sector [13].

There are various types of 3D printing, but they all share some typical characteristics: they begin with nearly nothing and build up the object layer-by-layer in three dimensions, and the object in all cases has to be designed on a software platform, then split into horizontal layers and finally printed layer-by-layer [14,15]. 

There are three techniques associated with extrusion-based three-dimensional printing: fused deposition modelling (FDM), semi-solid extrusion (SSE) and direct powder extrusion (DPE) [16,17,18].

All these 3D printing technologies necessitate the pre-processing of powders before they can be utilized for the 3D printing of tablets. This pre-processing step might impose restrictions on the selection of pharmaceutical-grade polymers. Specifically, FDM printing technology relies solely on hot melt extrusion (HME) as the pre-processing method to produce filaments loaded with drugs. The rheological behaviour and mechanical characteristics of specific materials might restrict their usage in FDM 3D printing [19,20,21]. However, researchers have documented a direct powder extrusion technology aiming at addressing challenges associated with current 3D printing methods [22,23]. In this method, pellets or milled extrudates are utilized with a direct powder extruder nozzle to produce the intended dosage forms through printing. This technology has been employed in creating paediatric dosage forms of the antiparasitic drug praziquantel and personalized dosage forms [24]. In a recent work, Dias-Torres et al. used micro-extrusion printing to investigate the effect of critical processing parameters on critical quality attributes for the printing of hydrochlorothiazide tablets [25]. According to Wang et al., micro-extrusion is superior compared to other 3D printing technologies, as its processing temperatures are lower but, most importantly, the technology can be used for the printing of semi-solid formulations and the development of paediatric dosage forms [23]. In addition, the technology can be implemented for point-of-care applications such as in hospitals or pharmacies due to the small footprint of the printers and the ease of replacing the printing cartridges. Moreover, Yang et al. have demonstrated the fabrication of 3D-printed hydrocortisone (HCT) (2.5, 5 and 7.5 mg) immediate-release tablets (all formulations exhibited >80% release in 40 min) for paediatric applications [26]. These printlets, which were comprised of Eudragit EPO, sodium stearyl fumarate, titanium dioxide, talc and triethyl citrate, were produced using FDM.

In the last five years, 3D printing technologies have been implemented in the development of paediatric dosage forms [27]. Scoutaris et al. initiated this trend by creating "candy-like" formulations resembling Starmix confectioneries, in which extruded filaments containing indomethacin and hypromellose (HPMC) acetate succinate effectively concealed the bitter taste of the drug [28]. Wang et al. recently developed 3D-printed donut-shaped tablets with caffeine citrate, achieving effective taste masking in paediatric immediate-release dosage forms by adjusting infill density [29]. Furthermore, Tagami et al. prepared a 3D-printed gummy drug formulation made of gelatine, HPMC, syrup, water and lamotrigine and achieved 85% drug release within 15 minutes [30]. Finally, Atabak et al. used micro-extrusion to fabricate paediatric chewable ibuprofen tablets and evaluated various polymers for their taste-masking purposes [10]. 

The objective of the present research was to develop an innovative micro-extrusion 3D printing method that eliminates the need for pre-processing steps while retaining the capability to regulate drug release through tablet design. Thus, sustained-release 5 mg hydrocortisone (HCT) paediatric mini-tablets for personalized use were fabricated. Only two excipients were used, Gelucire^®^ 44/14 and Precirol^®^ ATO 5, to avoid the possible adverse effects that accompany the conventional pharmaceutical manufacturing process, where a variety of excipients are used for blending, mixing, compression, release and packaging [31]. The printer utilized was a BIOX 3D printer. While this printer is widely used for bioprinting, to the best of our knowledge, it is the first time that Gelucire^®^ 44/14 and Precirol^®^ ATO 5 are used with this printer to fabricate solid tablet preparations [32]. In a similar study, Vithani et al. (2019) printed formulations of solid self-microemulsifying drug-delivery systems comprising Gelucire in grades 44/14 and 48/16, poloxamer as a surfactant and water-insoluble drug substances [33].

HCT is a corticosteroid that is used to treat paediatric patients with adrenocortical insufficiency [34]. The use of this native hormone is recommended over synthetic steroids (prednisolone and dexamethasone) due to its greater suppressive effect on growth [35]. According to the primary diagnosis, the HCT dose range is 7.5–15 mg/m^2^/day, given in three to four divided doses, with the first and slightly higher dose administered upon waking in the morning and the last dose administered 4 to 6 h before bedtime [36]. However, this regimen’s continuous administration poses challenges to patient compliance, particularly in children, who may need to disrupt their activities to adhere to their medication schedule, leading to discomfort and potential non-compliance. Orally administered HCT formulations are most commonly available in 10 mg tablets. As a result, in practice, caregivers are frequently required to administer a dose of 2.5 mg by manually cutting a 10 mg tablet into quarters [26]. In a recent study, it was shown that over half of the HCT 10 mg tablets tested did not meet the content criteria set by the United States Pharmacopoeia (USP) [37]. With regard to ensuring the safe administration of medications, children present a particularly difficult group of patients. They are more vulnerable to medication errors at every stage of the medicine management process due to the increased need for dose adjustment at regular intervals based on the age and weight of each individual [38]. Recognizing the above issues, there has been a growing interest in exploring the development of modified-release, 3D-printed tablets to reduce the frequency of dosing, potentially down to twice daily, and to personalise dosage according to individual needs in order to administer the exact correct dose for each child, achieving necessary therapeutic levels while reducing the side effects that come with potential overdosing. The high flexibility that 3D-printed medicines can offer is a promising tool in the treatment of the paediatric population [39].

## 2. Materials and Methods

Hydrocortisone (HCT) was obtained from Sigma-Aldrich (London, UK) and the excipients Gelucire^®^ 44/14 and Precirol^®^ ATO 5 from GATTEFOSSÉ (Lyon, France). The immediate-release 5 mg prototype of the commercially available HCT drug, named Hydrocortisone-SF (HCT–SF), was provided by SUN-FARM (Łomianki, Poland).

### 2.1. Three-Dimensional Printing 

**Figure 1:**
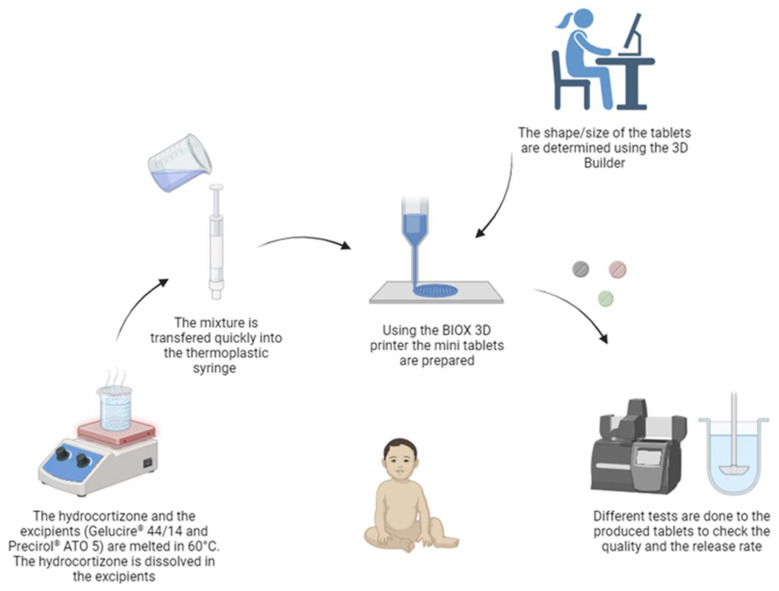
The methodology employed in crafting the 3D mini-tablets; created using BioRender.com https://www.biorender.com/ (accessed on 25 January 2024).

As depicted in Figure 1, initially, both the excipients (Gelucire^®^ 44/14 and Precirol^®^ ATO 5) and the drug were added into a beaker and heated at 60 °C under continuous stirring until the excipients completely melted and HCT was fully dissolved. The melted ink was then loaded into a thermoplastic syringe and inserted into the BIOX 3D printer for printing. A digital shape was designed using 3D Builder 20.0.4.0 and exported as .stl files. The dimensions determined were x = 3 mm, y = 3 mm and z = 2.5 mm. The .stl files were then transferred into the 3D printer via USB, and the printer parameters were adjusted directly through the BIOX 3D printer according to the formulations shown in Table 1. The size of the nozzle was 1.5 mm. 

### 2.2. Mini-Tablets’ Physical Characteristics

The mini-tablets were assessed for their weight and size (both width and height) with the use of a high-accuracy 4-digit scale and a vernier calliper, respectively. These measurements were conducted three times to ensure accuracy.

### 2.3. X-ray Powder Diffraction (XRPD)

X-ray powder diffraction patterns of bulk HCT and individually printed discs (23 × 1 mm) were recorded using aD8 Advance diffractometer (Brunker, Billerica, MA, USA) with Cu Kα radiation (40 kV, 40 mA) and a LynxEye silicon strip detector. A step size of 0.02° with a counting time of 0.3 seconds per step was used to collect data from 3–40° 2θ at a speed of 2° per min. The intensity and voltage applied were 15 mA and 40 kV. The angular range of data acquisition was 3–40° 2θ. 

### 2.4. Differential Scanning Calorimetry (DSC)

Thermal analysis of the HCT drug was performed using a DSC-3 (Mettler Toledo, Switzerland), which measures how much energy a sample absorbs or releases during heating or cooling. The samples (5 mg each) were accurately weighed in an aluminium pan before hermetically sealing them with the lid. The pure HCT, the two excipients (Gelucire^®^ 44/14 and Precirol^®^ ATO 5) and a physical mixture of all three were tested. Thermograms were taken within the temperature range of 25 °C to 250 °C, with a heating rate of 10 °C per minute and a continuous flow of nitrogen gas at a rate of 20 mL per minute. An aluminium pan without any content served as the reference.

### 2.5. Thermal Gravimetric Analysis (TGA)

Thermal gravimetric analysis of HCT was performed using a TGA (Q5000 (Thermal Instruments, Moraine, OH, USA) to investigate the thermal stability of HCT. An aluminium pan, initially weighing 52.24 mg, was loaded with the sample, resulting in a total weight of 59.76 mg. This means that 7.52 mg of the drug was added. Measurements were taken within the temperature rate of 25 °C to 350 °C with a heating rate of 10 °C per minute.

### 2.6. Dissolution Studies

The release of HCT from each formulation was tested using a USP II dissolution paddle apparatus (PharmaTest-D17, Hainburg, Germany) at 50 rpm and 37 ± 0.5 °C in aqueous dissolution media. For the first 2 h, a pH 1.2 solution was used to simulate the stomach environment, and thereafter, a buffer solution (pH 6.8) was used in order to adhere to the conditions in the small intestine. Samples (5 mL) were withdrawn at predetermined time intervals, filtered and then analysed using a UV-VIS spectrophotometer (uniSPEC 2 Spectrophotometer LLG Labware, Meckenheim, Germany), at λ_max_ = 248 nm. The dissolution experiments were performed in triplicate, and they resulted in the construction of graphs visualizing % release (mean ± SD) vs. time.

To determine the drug concentration in the mini-tablets, they were pulverized using a mortar and pestle, and subsequently, they were dissolved in 1 litre of deionized water while being continuously stirred with a magnetic stirrer for a period of 24 h. Samples of the solution were next passed through filters with a pore size of 0.45 μm (provided by Millipore Ltd. in Ireland). The drug concentration in the solution was assessed as above. The measurements were taken three times to ensure accuracy.

### 2.7. Mathematical and Statistical Analysis

The in vitro release data were fitted to the first- and zero-order release kinetics and Higuchi Korsmeyer–Peppas equations with GraphPad Prism 8 software (version 5.01) [40].

## 3. Results and Discussion

### 3.1. Printing Process

**Figure 2:**
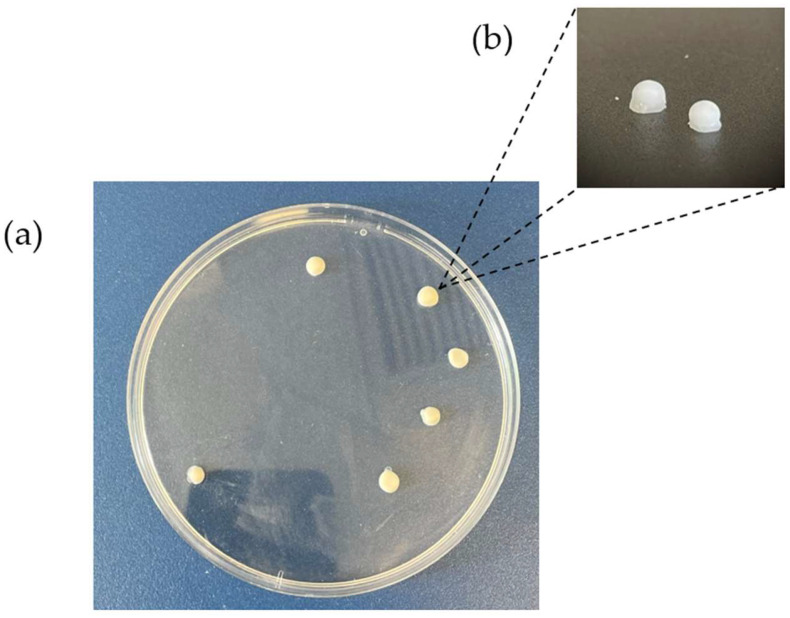
The 3D-printed HCT mini-tablets using the BIOX printer: (**a**) top view of the tablets, (**b**) side view of the tablets.

As previously mentioned, this study is the first time that the excipients Gelucire^®^ 44/14 and Precirol^®^ ATO 5 were employed for the fabrication of modified-release mini-tablets of HCT with the use of direct printing. Initially, a pneumatic syringe fabricated for the BIOX 3D printer was used, but it resulted in unsuccessful printing as the melted HCT/lipids mixture solidified quickly when it reached the nozzle. The syringe was unable to maintain the temperatures stable at 60 °C. Therefore, it was replaced with a metallic syringe designed for such materials which can uniformly maintain the required temperature throughout the process, allowing for the appropriate temperature and pressure settings to keep the mixture in a semisolid form and enabling extrusion and tablet production. The produced mini-tablets are shown in Figure 2.

One notable advantage of the generated tablets is that they are composed of lipophilic excipients, namely Precirol^®^ ATO 5 and Gelucire^®^ 44/14, which are not mixable with water-based foods, such as yoghurt and creams. As a result, their addition to those foods does not compromise the flavour or the composition of the tablets, thus making it easier for the children to take them. Also, due to the small size of the 3D-printed mini-tablets (1.5 × 6 mm), they can be easily added to food without crushing them or requiring any other alterations to the tablet’s shape or composition, so they can potentially be used as extemporaneous dosage forms. Additionally, Precirol^®^ ATO 5 is a known taste-masking agent, thereby ensuring that the produced mini-tablets do not present any unpleasant taste. These characteristics of the produced mini-tablets make them easier to administer to children, including infants and toddlers. 

The rationale behind using blends of Precirol^®^ ATO 5 and Gelucire^®^ 44/14 was to modify the drug release by varying the ratio of the two excipients. Gelucire^®^ 44/14 is a hydrophilic surfactant, which is used to increase the dissolution rate, while Precirol^®^ ATO 5 is a solid lipid suitable for sustained-release formulations. Hence, by adjusting the ratio of the two excipients, the HCT dissolution rates could be fine-tuned.

It can be observed that as the percentage of Precirol^®^ ATO 5 increases, the applied temperature and the pressure required to maintain the mixture in a semisolid state to facilitate extrusion increase accordingly. As a lipid-based excipient, Precirol^®^ ATO 5 typically has a higher melting point compared to Gelucire^®^ 44/14. As a result, by increasing the amount of Precirol^®^ ATO 5 in the mixture, the overall extrusion temperature must be raised as well. In addition, an increased proportion of Precirol^®^ ATO 5 increased the viscosity of the mixture, in turn requiring higher temperatures and greater pressure to achieve the flow characteristics desired for 3D printing. As layer adhesion is crucial for the structural integrity of 3D-printed objects, temperature and pressure must be adjusted as necessary to ensure that the material adheres well in each layer. This prevents the occurrence of defects or structural weaknesses in the final printed product. Moreover, as can be inferred from the above discussion, the excipients used are heat-sensitive; when exposed to temperatures exceeding 70 °C, there is a possibility of their composition being altered. Hence, it is crucial to carefully adhere to the manufacturer’s storage guidelines to ensure their stability.

### 3.2. Mini-Tablets’ Physical Characteristics

#### 3.2.1. X-ray Powder Diffraction (XRPD)

**Figure 3:**
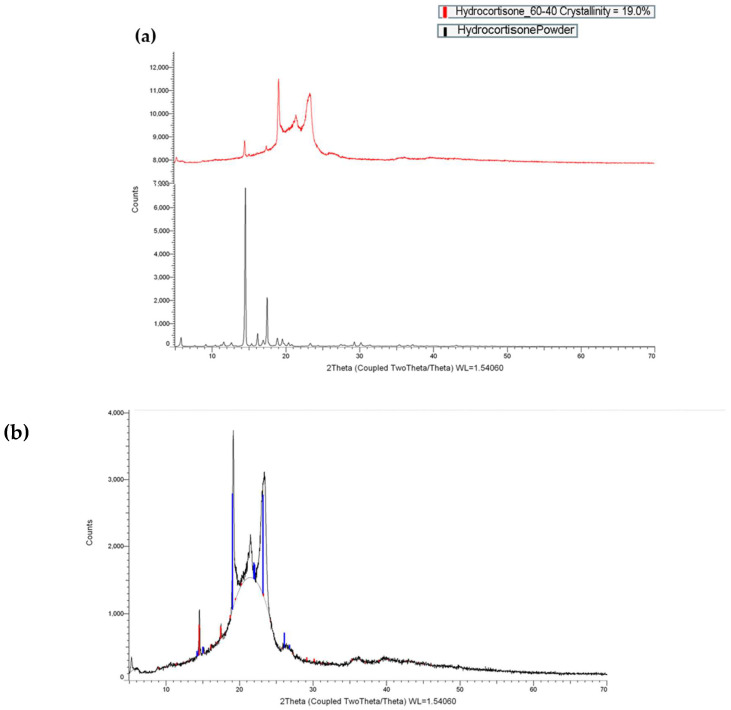
X-ray powder diffractograms of pure and printed drugs. (**a**) The formulation of 60/40 Gelucire^®^ 44/14 to Precirol^®^ ATO 5 with HCT. (**b**) Cumulative figure: the blue line depicts the small fraction of mono-, di- and triglycerides and mainly PEG-32 (MW 1500) which are present in Gelucire^®^ 44/14.

As shown in Figure 3, HCT was found to exist mostly in an amorphous state, and few crystalline peaks were noticed in the HCT post-printing. In the formulation with the 60/40 ratio of Gelucire^®^ 44/14 to Precirol^®^ ATO 5, 19% of the HCT was found in crystalline form. The HCT powder is 100% inherently crystalline but is transformed into an amorphous state during printing. This metamorphosis unfolds through the collaborative influence of lipids, specifically Gelucire^®^ 44/14 and Precirol^®^ ATO 5, known for their remarkable miscibility and compatibility with HCT. Despite the predominantly successful shift to the amorphous state, a notable 19% of the HCT retained its crystalline structure. A factor strongly contributing to this partial transformation is the low temperature at which the printing process was conducted. 

#### 3.2.2. Thermal Gravimetric Analysis (TGA)

**Figure 4:**
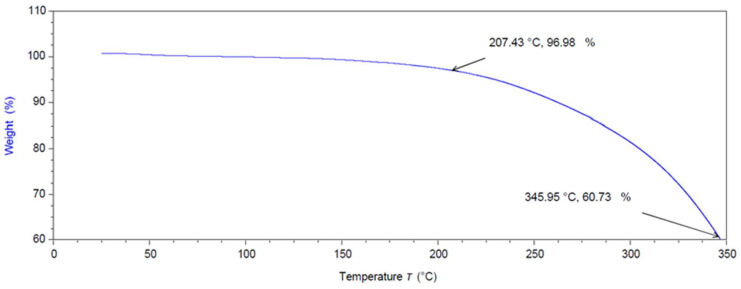
TGA thermogram of bulk hydrocortisone.

In the thermogravimetric analysis (TGA) test, measurements were conducted over a temperature range from 25 °C to 350 °C. As depicted in Figure 4, it was observed that 97 % of the drug remained intact at 207.43 °C. Notably, the melting point of HCT typically falls within the range of 215 °C to 220 °C.

However, a thermal degradation or decomposition of HCT of 36 % was observed at 345.95 °C. In all three ratios studied, the maximum printing temperature was 70 °C, which does not induce any HCT degradation according to the TGA findings.

#### 3.2.3. Mini-Tablets’ Dimensions and Weight Variations

In order to evaluate the mini-tablets, their weights and dimensions were measured (target dimensions: x = 3 mm, y = 3 mm, z = 2.5 mm) (Table 2). In general, all the formulations exhibited a marginally larger diameter than the target 6 mm (ranging from 6.14 mm to 6.35 mm). Simultaneously, when evaluating the height and weight, the observed variations across the three formulations remained consistent in alignment with the same measurements. 

#### 3.2.4. Differential Scanning Calorimetry (DSC)

DSC analysis was conducted on the bulk HCT, the lipid excipients (Precirol^®^ ATO 5 and Gelucire^®^ 44/14), the physical blend and the 3D-printed tablets to investigate the effect of the lipids and the printing process on the HCT’s physical state. 

**Figure 5:**
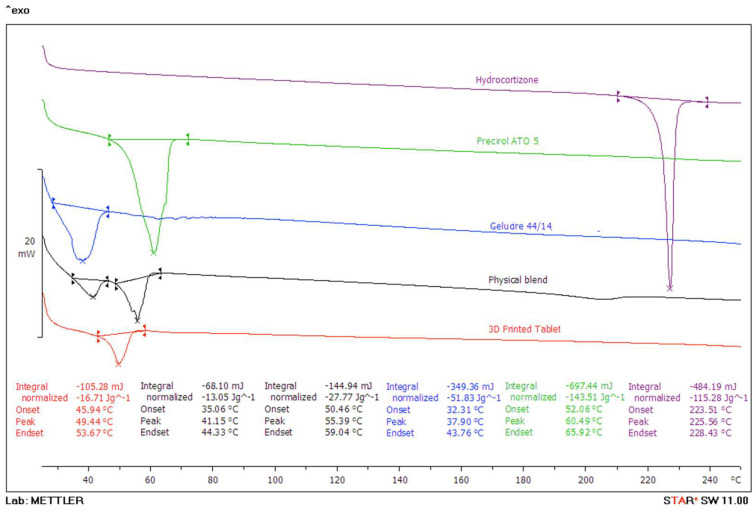
DSC thermogram of bulk hydrocortisone, the excipients (Precirol^®^ ATO 5 and Gellucire^®^ 44/14), and the physical blend.

As depicted in Figure 5, the obtained thermogram reveals a sharp, distinct endothermic peak of HCT at 225.56 °C, which is in agreement with previously reported values [41]. A sharp peak indicates a well-defined and narrow temperature phase transition range, with a distinct melting point to a more pure and crystalline substance. The printing process was conducted at temperatures varying between 60 and 70 °C, which are way below HCT’s melting point.

The excipients Precirol^®^ ATO 5 and Gelucire^®^ 44/14 both exhibit endothermic peaks at 60.49 °C and 36.41 °C, respectively, which are close to temperatures reported in similar studies [42,43]. Therefore, it can be considered that these endothermic peaks are attributed to the phase transition from a solid to a liquid state. Hence, the selected printing temperature range was adequate to melt the drug–excipient blends, allowing for successful extrusion.

Furthermore, Figure 5 shows that for the physical mixture, a very weak and broad melting endotherm appears at around 207 °C for HCT. The temperature shift and broad shape suggest that HCT is highly miscible with the lipids, which, in turn, solubilise the drug. It can be seen that a small fraction of HCT remained crystalline due to the high drug loading [44]. On the contrary, for the 3D-printed mini-tablets, the HCT melting endotherm completely disappears while the two melting endotherms of Precirol^®^ ATO 5 and Gelucire^®^ 44/14 merge into one onset peak at 45.99 °C. This suggests that HCT was further transformed into an amorphous state because of the second thermal processing. However, as the temperature increased during the DSC run, the HCT was fully solubilized in the pan, and it was not possible to detect any remaining crystalline quantities. Nevertheless, the combined usage of X-ray and DSC analysis showed that HCT is partially crystalline within the 3D-printed mini-tablets.

### 3.3. Drug Release Studies 

**Figure 6:**
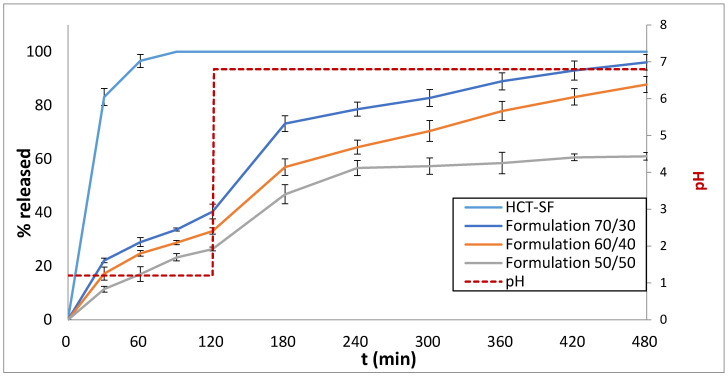
In vitro % release of HCT from 70/30, 60/40 and 50/50 formulations and the HCT-SF hydrocortisone tablets vs. time (min) at pH 1.2 (0–120 min) and at pH 6.8 (120–480 min). Results represent the mean value (n = 3, SD < 2).

As shown in Figure 6, the printed HCT mini-tablets showed sustained release profiles for all Precirol^®^ ATO 5 and Gelucire^®^ 44/14 compositions. During the first 2 h of the dissolution test, when the pH was 1.2 to simulate the gastric environment, the release of HCT from the 3D mini-tablets (70/30, 60/40 and 50/50) was <40%. After the first 2 h, the release of HCT was not affected by the change in pH to 6.8 (representing the intestinal environment). In particular, the increase in the Gelucire^®^ 44/14 content in the formulation led to a more prolonged HCT release (t_90%_ for the 70/30 ratio was 380 min; t_90%_ for 60/40 was 464 min; t_90%_ for 50/50 was not applicable, as a 90% release was not attained after 8 h). The dissolution rates of HCT were compared with currently marketed HCT tablets, namely HCT-SF. The obtained HCT release from the HCT-SF tablets was immediate, and more than 80% was released within the first 30 min. The results demonstrate that, in contrast to the commercially available product, HCT was released in a more sustained manner from the 3D mini-tablets. This is due to the use of Precirol^®^ ATO 5 and Gelucire^®^ 44/14 blends, which allowed the drug release to be modified by varying the ratio of the two excipients.

Our results align with those from a similar study conducted by Ayyoubi et al. (2023), who developed sustained-release 3D-printed tablets using FDM and drug-loaded filaments produced via HME [45]. Another study, conducted by Parulski et al. (2023), examined the fabrication of sustained-release tablets loaded with HCT using FDM, but with shorter release rates of up to 2 h [46]. Similarly, Yang et al. (2023) developed immediate-release HCT tablets using FDM, but the detected impurities were close to the required threshold [26].

In our case, the printing process is simpler as it does not require any HME processing and the mini-tablets can be directly printed. Furthermore, we introduce the use of lipid excipients to aid in the development of sustained-release formulations without the need for complex formulations as used in the aforementioned studies or to thermally process the HCT formulations more than once. The use of FDM is based on the printability of the filaments, requiring detailed studies prior to printing, thus making the process more time-consuming. 

Table 3 summarizes the release mechanisms for the 3D-printed mini-tablets. As the HCT-SF generic tablets presented immediate release, the data did not fit the release kinetic models used in this study. Based on the regression coefficients (R^2^) of Table 3, the release mechanism for the 3D-printed mini-tablets follows the Higuchi model. Regarding the value n of the Korsmeyer–Peppas equation, it is shown that all the fabricated formulations follow anomalous diffusion release kinetics (0.45 ≤ n ≤ 0.89). 

Bearing in mind that HCT is typically administered to children in three to four immediate-release doses, compliance among young patients is low. Conversely, by administering 3D-printed mini-tablets with customized release profiles (potentially at different strengths) to young patients, the doses per day are minimized where required in comparison to the commercially available formulation, thus enhancing compliance among children [47]. In addition, by limiting the frequency of the doses, adverse effects are minimized. Another major advantage of the sustained-release 3D-printed mini-tablets is that they can improve HCT’s clinical performance by imitating the body’s cortisol levels according to the circadian rhythm. As mentioned earlier, Ayyoubi et al. and Parulski et al. developed 3D-printed sustained-release HCT tablets based on this same concept, where the release profiles could be customized to meet individuals’ cortisol levels by altering the filament composition [45,46].

## 4. Conclusions

HCT is frequently employed in paediatric patients to address a range of conditions, often necessitating chronic administration. Given the substantial diversity among children, both inter-individually and in terms of their constantly evolving physiological profiles, including weight and personal characteristics, the development of personalized therapeutic formulations is imperative.

In this study, we implemented micro-extrusion 3D printing for the preparation of paediatric mini-tablets. The printing process was optimized by adjusting the applied temperature and pressure, which led to consistent printability with high accuracy regarding the mini-tablets’ shapes and sizes. By tuning the composition of the printable inks, it was possible to produce mini-tablets with customizable sustained HCT release rates. Since infants and children struggle to swallow some pills, the developed 3D-printed mini-tablets incorporating lipophilic excipients Precirol^®^ ATO 5 and Gelucire^®^ 44/14 could be used as extemporaneous formulations in food without altering the taste or the composition. Furthermore, the sustained release of HCT could potentially lead to a reduction in the number of required daily doses. This, in turn, would enhance compliance and mitigate adverse side effects. Currently, we are extending the application of micro-extrusion to the development of paediatric dosage forms for a range of drug substances. Overall, micro-extrusion is advantageous over other 3D printing technologies, as the printing of the dosage forms requires fewer steps while the small footprint offers enhanced printing capabilities.

## Figures and Tables

**Table 1:** The parameters employed for the three different ratios of Gelucire^®^ 44/14 to Precirol^®^ ATO 5 during the printing process.

Excipients Ratio	Nozzle Temp. (°C)	Plate Temp. (°C)	Infill Density (%)	Layer Height (mm)	Pressure (kPa)	Speed (mm/s)
(70/30) Gelucire^®^ /Precirol^®^	60.0	9.0	60.0	1.5	44.0	4.0
(60/40) Gelucire^®^ Precirol^®^	65.0	9.0	60.0	1.5	59.0	4.0
(50/50) Gelucire^®^/Precirol^®^	70.0	9.0	60.0	1.5	74.0	4.0

**Table 2:** Physical characteristics of printed mini-tablets (n = 10).

Excipients Ratio	Width (mm)	Height (mm)	Weight (mg)
(70/30) 70% Gelucire^®^ 44/14 and 30% Precirol^®^ ATO 5	6.23 ± 1.03	2.5 ± 0.255	25 ± 3.08
(60/40) 60% Gelucire^®^ 44/14 and 40% Precirol^®^ ATO 5	6.35 ± 1.21	2.5 ± 0.230	25 ± 3.25
(50/50) 50% Gelucire^®^ 44/14 and 50% Precirol^®^ ATO 5	6.14 ± 1.11	2.5 ± 0.250	25 ± 3.13

**Table 3:** Mathematical models (* these values could not be calculated).

Formulation	Zero-Order			First-Order		Higuchi		Korsmeyer–Peppas		
	R^2^	Y_0_	Κ_0_	R^2^	Y_1_	R^2^	Κ_H_	R^2^	Κ_HP_	*n*
HCT-SF	*	*	*	*	*	*	*	*	*	*
Formulation 70/30	0.89	24.06	0.17	0.80	35.44	0.94	4.58	0.92	2.37	0.63
Formulation 60/40	0.95	17.37	0.16	0.87	28.16	0.96	3.95	0.95	1.86	0.64
Formulation 50/50	0.85	15.30	0.12	0.74	23.49	0.91	3.02	0.90	2.23	0.55

## Data Availability

All data generated during this study are included in this published article.
